# Early-Life Stress Alters Synaptic Plasticity and mTOR Signaling: Correlation With Anxiety-Like and Cognition-Related Behavior

**DOI:** 10.3389/fgene.2020.590068

**Published:** 2020-12-14

**Authors:** Anfeng Wang, Xiaojuan Zou, Jiajia Wu, Qingyu Ma, Naijun Yuan, Fengmin Ding, Xiaojuan Li, Jiaxu Chen

**Affiliations:** ^1^School of Basic Medical Science, Hubei University of Chinese Medicine, Wuhan, China; ^2^Formula-Pattern Research Center, School of Traditional Chinese Medicine, Jinan University, Guangzhou, China; ^3^School of Traditional Chinese Medicine, Beijing University of Chinese Medicine, Beijing, China

**Keywords:** early-life stress, maternal separation, synaptic plasticity, mTOR, s6

## Abstract

Early-life stress (ELS) predisposes individuals to psychiatric disorders, including anxiety and depression, and cognitive impairments later in life. However, the underlying molecular mechanisms are not completely understood. Developmental deficits in hippocampal synaptic plasticity are among the primary detrimental alterations in brain function induced by ELS. Impaired synaptic plasticity is usually accompanied by decreased synaptic proteins, such as postsynaptic density 95 (PSD95) and synaptophysin, which are important for synaptic function. The mTOR signaling pathway plays a vital role in regulating protein translation, and mTOR activation is functionally associated with synaptic protein synthesis. In the present study, we observed whether ELS impacts synaptic protein synthesis and mTOR signaling, which is involved in synaptic plasticity. Herein, we established a maternal separation (MS) and chronic restraint stress (CRS) model and evaluated anxiety-like behavior and cognitive function (e.g., learning and memory) in adulthood through behavioral examination and analyzed hippocampal expression levels of PSD95 and synaptophysin. To explore whether the mTOR signaling pathway was associated with ELS, we also examined the activity of mTOR and s6. The behavior tests indicated that maternally separated mice showed increased anxiety-like behavior and cognitive impairments. PSD95 and synaptophysin mRNA and protein expression levels were decreased in the hippocampus, and phosphorylated mTOR and phosphorylated s6 were significantly decreased in maternally separated mice vs. those not exposed to MS. Our data demonstrate that MS impairs synaptic plasticity and inhibits mTOR signaling, specifically via s6. Therefore, we speculate that ELS decreased synaptic plasticity via the inhibition of the mTOR pathway in the hippocampus, which may underlie vulnerability to stress and mental disorders in adulthood.

## Introduction

Early-life stress (ELS), such as experiencing emotional neglect, physical abuse or traumatic events, can lead to long-lasting changes in neuronal physiology (Fumagalli et al., 2007). Clinical and epidemiological studies have suggested that individuals exposed to early adverse experiences have an increased risk of mental disorders, including anxiety and depression, and cognitive deficits (Harkness et al., 2006; Carr et al., 2013; Ménard et al., 2016). Maternal separation (MS) is an animal model of ELS that has been widely used in recent decades (Vetulani, 2013; Kambali et al., 2019). Several studies have reported that MS negatively impacts brain function, resulting in increased anxiety- and depressive-like behaviors and impaired cognitive function (Boccia et al., 2007; Desbonnet et al., 2010; Nishi et al., 2014). Thus, it has been suggested that adverse experiences in early life may induce vulnerability to the effects of stress later in life. However, the mechanisms by which this occurs are still not completely understood.

The regulation of synaptic plasticity is closely related to the induction of mental disorders. Based on existing evidence, ELS affects synaptic function and impairs synaptic plasticity (Sousa et al., 2014; Jeanneteau and Arango-Lievano, 2016). It has been previously reported that decreased postsynaptic density 95 and synaptophysin levels were found in MS rats (Martisova et al., 2013; Dandi et al., 2018). Mammalian target of rapamycin (mTOR) is a protein kinase that belongs to the phosphatidylinositol 3-kinase-related kinase protein family, which integrates signals from neuronal activity, growth factors, and nutrient levels to regulate the initiation of protein translation (Abelaira et al., 2014). Interestingly, it has been reported that mTOR is involved in translation control and long-lasting synaptic plasticity (Hoeffer and Klann, 2010). Previous studies have shown that reduced mTOR signaling function could result in decreased synthesis of synaptic proteins (Duman et al., 2016). Dysregulation of mTOR can lead to various mental illnesses (Hoeffer and Klann, 2010). Clinical studies have also found deficits in the mTOR signaling pathway in subjects with major depressive disorder (Jernigan et al., 2011), and activation of the mTOR pathway is related to antidepressant actions (Suo et al., 2013). However, it is poorly understood whether ELS affects mTOR signaling.

In the present study, we evaluated the effects of MS and subsequent chronic restraint stress (CRS) on behavior and synaptic proteins in the hippocampus of mice. In addition, we investigated whether the changes in behavior and synaptic plasticity were accompanied by inhibition of the mTOR pathway in the hippocampus.

## Materials and Methods

### Animals

All protocols involving experimental animals were reviewed and approved by the Institutional Animal Care and Use Committee at Jinan University (approval No. IACUC-20190702-03). C57BL/6J female mice that were 15 days pregnant were purchased from the Experimental Animal Center at Guangzhou University of Chinese Medicine and individually housed until delivery. The day of delivery was considered postnatal day zero (postnatal day 0; PND0). Male pups were used in the present study. All animals were housed in a room at a temperature of 22 ± 2°C and 40–45% humidity and on a 12-h light/dark cycle and they were allowed free access to chow and water. Behavioral testing began at PND60. At PND69, all mice were euthanized, and hippocampal tissues were separated on 4°C ice for real-time PCR and western blotting experiments.

### Experimental Design

**FIGURE 1:**
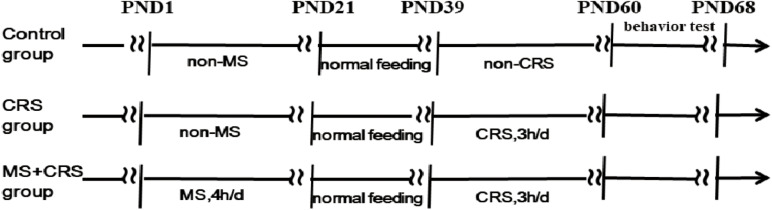
Schematic representation of the experimental design.

The male pups were assigned to three groups of 9–10 mice per group. The control group consisted of non-separated, non-restrained mice. The CRS group consisted of non-separated, restrained mice. The MS + CRS group consisted of maternally separated and restrained mice (Figure 1 for the experimental design).

### Maternal Separation and Chronic Restraint Stress Procedure

The MS procedure was initiated at PND1, and the protocol was performed as previously described (McCoy et al., 2016; Park et al., 2018), with slight modifications. From PND1 to PND21, the pups were removed from home cages to separate them from their dams and placed in another separate room for 4 h (8:00–12:00) a day. Following a 4-h separation period, the pups were then returned to their original cages and cared for by their mothers. Non-separated pups stayed with their dams and remained undisturbed in their cages (except for cage cleaning once every 3 days) until PND22. All litters were weaned and separated by sex at PND22, and only male pups were used in the current study.

At PND39, the pups in the CRS and MS + CRS groups were subjected to CRS according to a previously reported protocol (Campos et al., 2013; Lee et al., 2019). Briefly, the mice were placed in 50-mL conical tubes with air holes drilled into the sides to restrict their movements for 3 h (9:00–12:00 h) daily for 3 weeks.

### Behavioral Testing

To detect changes in behavior and cognition (e.g., learning and memory), mice were subjected to behavioral tests that were performed the day after CRS ended (Figure 1 for timeline). The mice were placed in the test room to adapt to the environment for more than 1 h before each behavioral test. Ethovision (v.14.0, Noldus Information Technology) was used to record and analyze mouse behavior.

#### Open Field Test

Anxiety-like behavior and locomotor activity were assessed in the open field test (OFT) (Lau et al., 2008). The open-field apparatus used in this study was a square arena (50 cm× 50 cm) surrounded by 40 cm high black walls. The arena’s white floor was divided into 25 equal squares by black lines. During the test, the mice were placed at the center of the arena. In this test, the mice were individually placed at the center of the arena and allowed to freely explore the apparatus for 5 min. Between tests, the apparatus was cleaned with 70% ethanol to prevent the smell of the previous mouse influencing the subsequently tested mice. The time spent in the center area and total distance moved were evaluated.

#### Elevated Plus Maze

The elevated plus maze (EPM) test was performed to measure anxiety-like behavior in the current study. The maze consisted of two open arms (67 cm × 7 cm) and two enclosed arms (67 cm × 7 cm × 14 cm), which were placed 65 cm above the floor. Each mouse was placed in the central zone of the maze and left to freely explore for 5 min. Decreased time spent in and number of entries into the open arms suggest higher levels of anxiety (Walf and Frye, 2007; Dandi et al., 2018). Seventy percent ethanol was used to clean the maze as in the OFT. We analyzed the number of entries into the open arms and the ratio of open arm time (open arms time/total arms time).

#### Novel Object Recognition Task

The novel object recognition task (NORT) was performed to investigate non-spatial learning and memory and was carried out according to the procedure described in previous studies (Kruk-Slomka et al., 2014; Lueptow, 2017). Specifically, the test was performed in an open-field apparatus (50 × 50 × 40 cm^3^) and consisted of three trials:habituation trial, training trial and testing trial. In the habituation trial, the mouse was placed into the open field without objects and allowed to explore for 5 min. After 24 h, training and testing trials were performed. First, two identical objects (two cylindrical boxes) were placed at the left and right back corners of the apparatus. Then, the mice were placed into the apparatus and allowed to explore for 10 min for the training trial. After an hour, one cylindrical box was replaced with a cube box for the testing trial, and the mice were allowed to freely explore for 10 min. Seventy percent ethanol was used to remove the olfactory cues of the object. Because animals are naturally fond of novelty, if the mouse remembered the familiar objects, it will spend more time with the novel object. Therefore, decreased time spent with the novel object suggested cognitive impairment. The exploration time of the two objects was recorded in the testing trial. The recognition index (RI) was calculated as 100% × time with the novel object/time with both objects.

#### Morris Water Maze

In the present study, the Morris water maze (MWM) was used to assess spatial learning and long-term memory with spatial learning and probe trials. The test was carried out according to the method described by Weitzner et al. (2015) with some modifications and conducted in 5-day blocks. The maze was a circular open pool (approximately 1.2 m in diameter) and divided into four quadrants: northeast (NE), northwest (NW), southeast (SE), and southwest (SW). The escape platform was placed in the center of the NW quadrant and submerged 0.8 cm below the water surface. Four pictures of different shapes were pasted on the walls of the four quadrants as maze cues. The acquisition trials lasted for 4 days. Each mouse received four trials per day and were released at four different positions in a semirandomized way. On each trial, the mice were placed in the water facing the pool wall and allowed to swim until locating the escape platform or until a maximum of 60 s. Upon finding the platform, the mice remained on the platform for 15 s before removal from the pool and placement in the cage. If a mouse did not locate the platform within 60 s, it was guided to the platform and remained there for 15 s before being returned to its cage. The latency to escape (time to reach the platform) and swim speed were measured for each mouse. On the fifth day, the probe trial was administered without a platform in the pool. The mouse was released from a point in the opposite quadrant (SE) and allowed to explore for 60 s. The time spent in the target quadrant and the number of crossings over the previous position of the escape platform were recorded.

### Real-Time PCR

The expression of PSD95 and synaptophysin in the hippocampus of mice was measured by RT-PCR. Total RNA was isolated from hippocampal tissues using TRIzol (Invitrogen, United States), and its concentration was detected by spectrophotometry (Eppendorf, Germany). According to the instructions of the Revertaid First Strand cDNA Synthesis Kit (Thermo Fisher Scientific, Waltham, MA), the total RNA concentrations were normalized to 20 μl before reverse transcription into cDNA. Sequences for the primers are shown in Table 1 and were designed by Sangon Biotech Co., Ltd. (Shanghai, China). Power SYBR^®^Green PCR Master Mix (Thermo Fisher Scientific) was used for fluorescence qPCR to amplify samples using the following cycling parameters: 95°C for 30 min and 40 cycles of 95°C for 5 s and 60°C for 30 s. All results are expressed relative to glyceraldehyde-3-phosphate dehydrogenase (GAPDH) in the present study and performed by Bio-Rad CFX Manager 1.1 (Bio-Rad, United States).

**TABLE 1:** Primer sequences used in RT-qPCR analysis.

Gene		Primer sequence
PSD-95	Forward	5′-TCCAGTCTGTGCGAGAGGTAGC-3′
	Reverse	5′-CAGGGAGCGGGGACGGATG-3′
Synaptophysin	Forward	5′-AGTACCCATTCAGGCTGCAC-3′
	Reverse	5′-CCGAGGAGGAGTAGTCACCA-3′
GAPDH	Forward	5′-GGCAAGTTCAATGGCACAGT-3′
	Reverse	5′-AAAGTGGAGGAATGGGAGTT-3′

### Western Blot

The expression levels of PSD95, synaptophysin, mTOR, p-mTOR, s6, and p-s6 proteins in the mouse hippocampus were tested by Western blot analysis. Total protein from hippocampal tissues was extracted in RIPA buffer with proteinase and phosphatase inhibitors. A BCA protein assay kit was used to examine the concentration of total protein, which was adjusted to 2 mg/ml. Proteins were resolved by 8 or 10% SDS-PAGE gel electrophoresis and transferred to PVDF membranes. After blocking with 5% skim milk or 90 min and then washing 2 × 10 min in TBST buffer, the membranes were incubated overnight at 4°C with primary antibody. The next day, the membranes were washed with TBST buffer and then incubated for 60 min with secondary antibody. Bands were developed by enhanced chemiluminescence reagent (Millipore, Billerica, MA, United States) and subsequently visualized by an imaging system (Bio-Rad, California, United States). Finally, Image J was used to quantify the integrated gray densities of each band. The primary and secondary antibody used in Western blotting in the present study are listed in Table 2.

**TABLE 2:** Primary and secondary antibody used in Western blotting.

Antigen	Host	Manufacturer	Dilution	Catalog#
PSD95	Rabbit	CST	1:1,000	3409
Synaptophysin	Rabbit	CST	1:1,000	36406
Phospho-mTOR (Ser2448)	Rabbit	Abcam	1:5,000	Ab109268
mTOR	Rabbit	CST	1:1,000	2983
Phospho-s6 (Ser240/244)	Rabbit	CST	1:1,000	5364
S6	Rabbit	CST	1:1,000	2217
Bate actin	Rabbit	Affinity	1:10,000	AF7018
Goat anti-rabbit IgG (H + L)	Goat	ZSGB-BIO	1:7,000	ZB-2010

### Statistical Analysis

All data were analyzed using SPSS 20.0 (IBM software) and presented as the mean ± SEM. When the difference was at the level of *P* < 0.05, the data were accepted as statistically significant. Two-way repeated measures analysis of variance (ANOVA) was used to analyze latency to escape (time to reach the platform) and swim speed in the MWM. The remaining data were assessed using one-way ANOVA and then using *post-hoc* Tukey tests.

## Results

### Analysis of Anxiety-Like Behaviors

**FIGURE 2:**
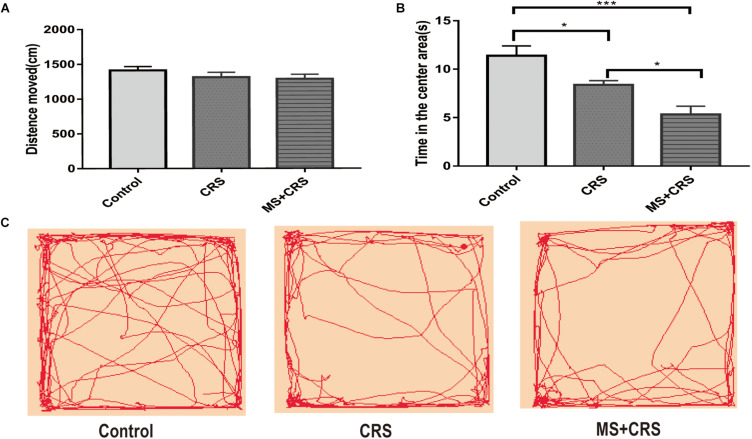
Behavioral changes during the open field test (OFT). **(A)** Means of distance moved and **(B)** Time in the center area. **(C)** Representative video tracking images during 5 min in the OFT. Data are presented as the mean ± SEM. *n* = 9–10 mice in each group. *P < 0.05, ***P < 0.001.

In this study, the OFT and EPM task, two reliable behavioral tests of anxiety, were used to evaluate emotionality. As shown in Figures 2A,B, in the OFT, the results showed that the CRS and MS + CRS groups were significantly different compared with the control group [*F*(2, 26) = 13.55, *P* < 0.05; *P* < 0.001] regarding the time spent in the central squares. In particular, both the CRS and MS + CRS mice spent less time in the center squares than the control mice. Moreover, the time was significantly lower in the MS + CRS group than in the CRS group (*p* < 0.05). However, the distance that the CRS and MS + CRS groups moved was not significantly different from that in the control group, which indicated that neither CRS nor MS + CRS affected the total distance moved of the mice in the OFT. Figure 2C shows tracking images.

**FIGURE 3:**
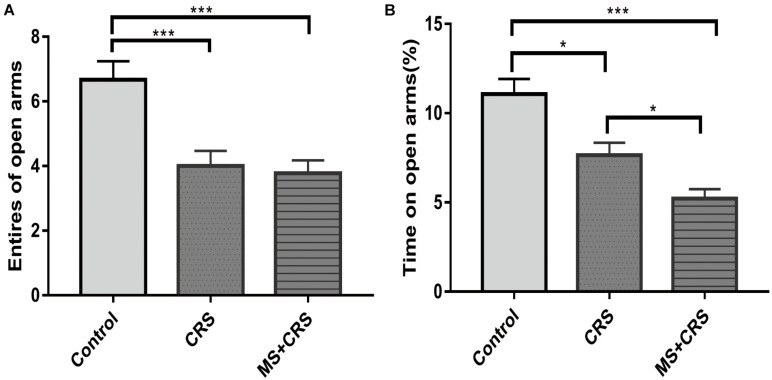
Behavioral changes in the elevated plus maze (EPM). **(A)** Entries into open arms. **(B)** Time spent in the open arms expressed as a ratio of time spent in both open and closed arms of the EPM. Data are presented as the mean ± SEM; *n* = 9–10 mice in each group. *P < 0.05, ***P < 0.001.

Behavioral changes for the three groups observed in the EPM test are presented in Figure 3. As shown in Figures 3A,B, both the CRS and MS + CRS groups had a decreased number of entries and percentage of time spent in the open arms [*F*(2, 25) = 10.611, *P* < 0.01, *P* < 0.001; *F*(2, 25) = 17.748, *P* < 0.05, *P* < 0.001] compared with the control group. Moreover, the percentage of time spent in the open arms was lower in the MS + CRS group than in the CRS group (*P* < 0.05), but no significant difference between the above two groups was observed in the number of entries into the open arms (*P* > 0.05). These results indicated that the mice in the MS + CRS group displayed more anxiety-like behavior than those in the CRS group.

### Analysis of Learning and Memory Behavior

**FIGURE 4:**
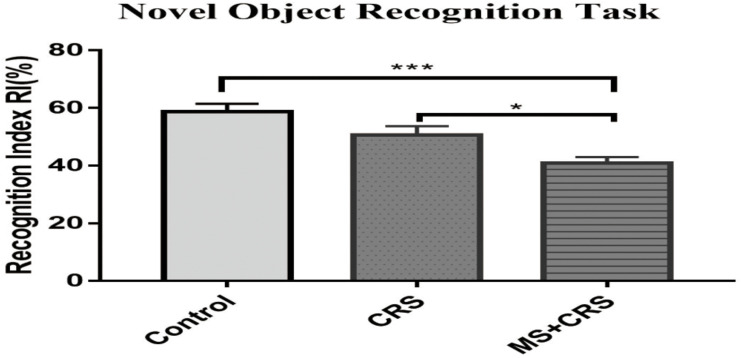
Behavioral changes in the novel object recognition task (NORT). Performance in the NORT shown by the recognition index. Error bars, SEM; *n* = 9–10 mice in each group. *P < 0.05, ***P < 0.001.

To examine whether MS affects learning and memory, the MWM test and NORT were used in the current study to evaluate memory and spatial and non-spatial learning. As shown in Figure 4, the RI value in the MS + CRS mice was significantly different from that in the control mice [*F*(2, 26) = 10.797, *P* < 0.001] in the NORT. Specifically, *post-hoc* comparisons revealed that the difference in exploration time between the novel object and the familiar object was lower in the MS + CRS group than in the control group (*P* < 0.05). However, no significant difference in the RI value was observed between the CRS and control groups (*P* > 0.05). The results indicated that MS disrupted non-spatial learning and memory processes.

**FIGURE 5:**
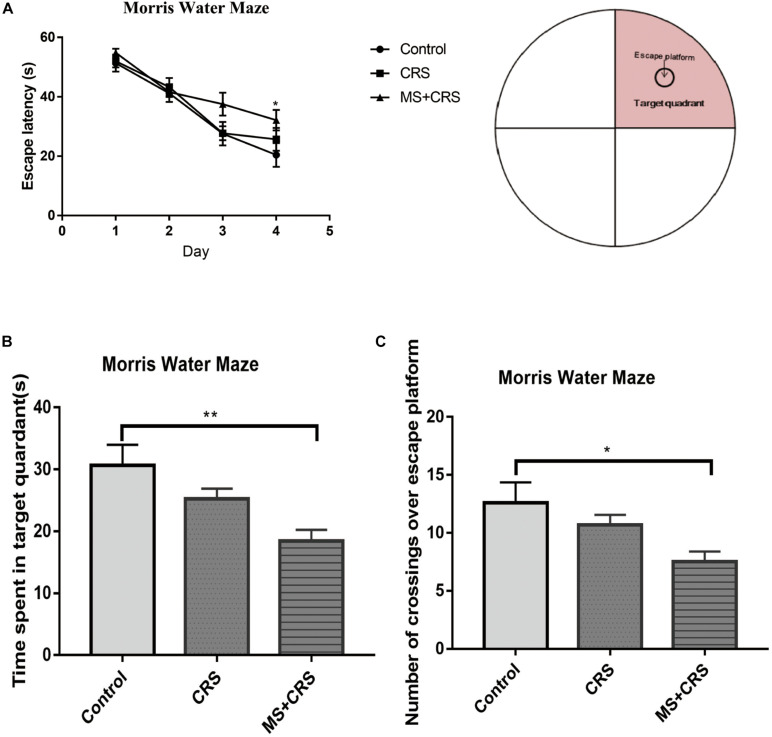
Behavioral changes in the Morris water maze (MWM). **(A)** Escape latency time during the acquisition trial. **(B)** Time spent in the target quadrant during the probe trial. **(C)** Number of crossings over the previous location of the escape platform during the probe trial. Error bars, SEM; *n* = 9 mice in each group. *P < 0.05, **P < 0.01.

In the MWM, two-way repeated measures ANOVA showed that the latency to escape on the hidden platform significantly differed between group and training days [group: *F*(2, 24) = 4.560, *p* < 0.05]; training days [*F*(3, 72) = 43.981; *p* < 0.001]. As shown in Figure 5A, the escape latency for mice in all experimental groups was shortened with the passage of training days. On the fourth day of training, the time to reach the platform was significantly higher in the MS + CRS group than in the control group (*p* < 0.05). The latency in the CRS mice showed an increasing tendency, but the difference from the control group was not statistically significant. These results indicated that spatial learning abilities were impaired in the mice in the MS + CRS group but not the CRS group. On the fifth day, a probe trial was performed. The results are shown in Figures 5B,C. We observed that the time spent in the target quadrant [*F*(2, 26) = 6.443, *p* < 0.01] and the number of crossings over the previous location of the escape platform [*F*(2, 26) = 4.21, *p* < 0.05] were both significantly lower in the MS + CRS group than in the control group, but no difference was observed between the CRS and control groups.

### Effects on Hippocampal PSD95 and Synaptophysin mRNA and Protein Levels

**FIGURE 6:**
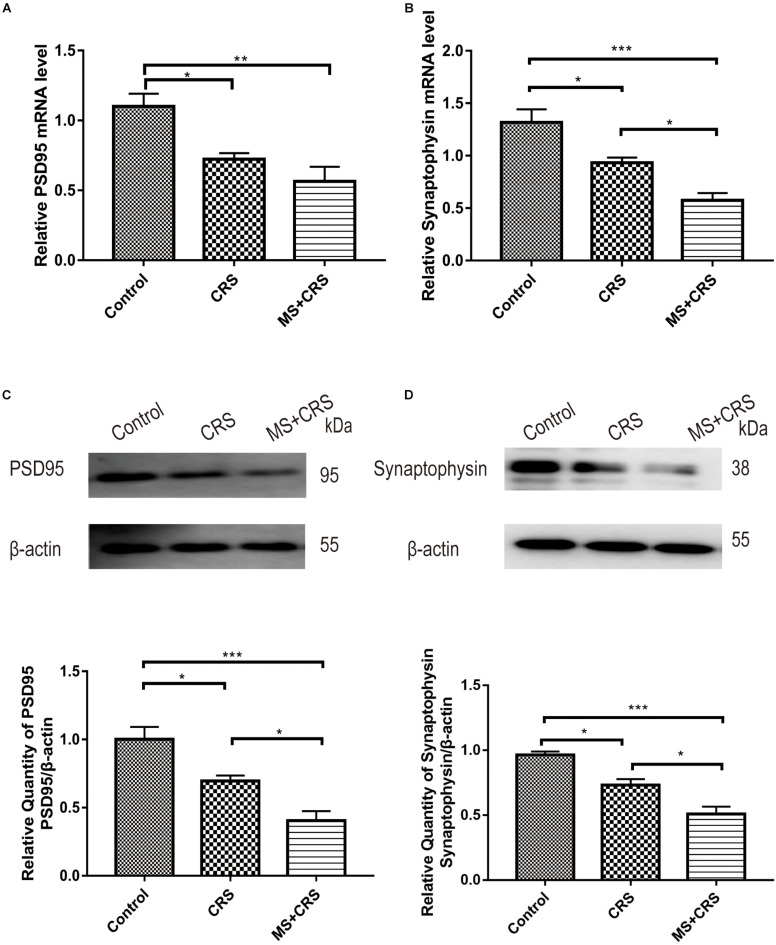
PSD95 and synaptophysin mRNA and protein expression levels in the hippocampus. mRNA expression levels were measured by RT-qPCR, and the experiment was repeated twice. Quantitative analysis was normalized to GAPDH. Protein expression levels were determined by Western blotting analysis. **(A)** PSD95 mRNA expression (*n* = 4). **(B)** Synaptophysin mRNA expression (*n* = 4). **(C)** PSD95 protein expression (*n* = 4). **(D)** Synaptophysin protein expression (*n* = 4). Gel images from Western blotting. Data are presented as the mean ± SEM. *P < 0.05, **P < 0.01, ***P < 0.001.

The PSD95 and synaptophysin mRNA levels in the hippocampus were measured by quantitative RT-PCR. The relative target gene mRNA levels in the groups are shown in Figure 6. As shown in Figures 6A,B, the expression levels of PSD95 mRNA in the hippocampus were lower in the mice in the CRS and MS + CRS groups than in the mice in the control group [PSD95: *F*(2, 9) = 10.856, *P* < 0.05, *P* < 0.01; synaptophysin: *F*(2, 9) = 17.920, *P* < 0.05, *P* < 0.001]. Tukey’s *post-hoc* tests revealed that the levels of synaptophysin mRNA in the MS + CRS group were significantly lower than those in the CRS group (*p* < 0.05). However, the levels of PSD95 mRNA between the CRS and MS + CRS groups showed no significant difference; although the results from the MS + CRS group showed a trend toward a reduction compared with the CRS group, this difference did not reach statistical significance (*P* > 0.05).

Changes in PSD95 and synaptophysin protein expression are shown in Figures 6C,D. One-way ANOVA revealed that the CRS and MS + CRS groups had significantly lower PSD95 and synaptophysin protein levels than the control group [PSD95: *F*(2, 9) = 18.441, *P* < 0.05, *P* < 0.001; synaptophysin: *F*(2, 9) = 26.074, *P* < 0.05, *P* < 0.001]. Moreover, Tukey’s *post-hoc* test demonstrated that the MS + CRS group had significantly lower PSD95 and synaptophysin protein levels than the CRS group (*P* < 0.05, both). Whole-gel images from the Western blotting are provided in Figures 6C,D.

### Effects on the Hippocampal mTOR-s6 Pathway

**FIGURE 7:**
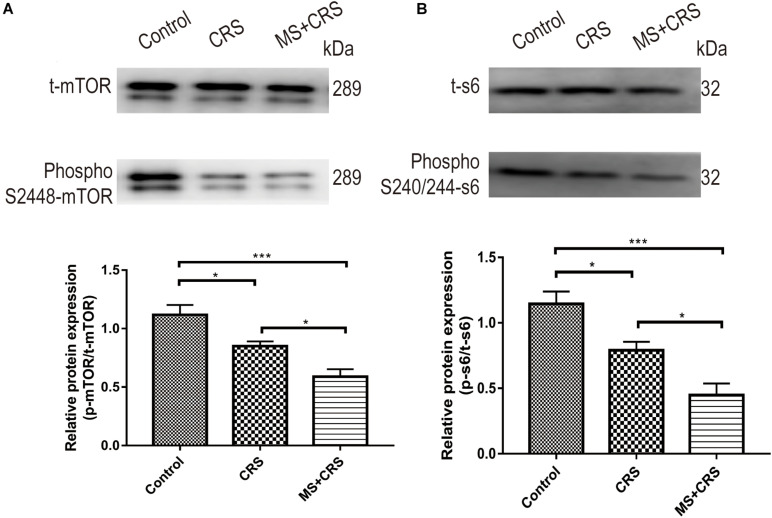
p-mTOR/t-mTOR and p-s6/t-s6 protein expression levels in the hippocampus. **(A)** p-mTOR/t-mTOR protein expression (*n* = 4). **(B)** p-s6/t-s6 protein expression (*n* = 4). Gel images from Western blotting. Data are presented as the mean ± SEM. *P < 0.05, ***P < 0.001.

We observed an appreciable reduction in phospho-mTOR (S2448) and phospho-s6 (S240/244) in the CRS and MS + CRS groups compared with the control group [phospho-mTOR/t-mTOR: *F*(2, 9) = 16.528, *P* < 0.05, *P* < 0.001; phospho-s6/t-s6: *F*(2, 9) = 17.193, *P* < 0.05, *P* < 0.001], but the total mTOR and s6 levels remained unaltered. Reduced phosphorylated levels of mTOR and s6 protein in the CRS and MS + CRS mice indicated that the mTOR-S6 pathway was inhibited. As further evidence, the results showed that the levels of phospho-mTOR and phospho-s6 were significantly lower in the MS + CRS group than in the CRS group (*P* < 0.05) (Figure 7).

## Discussion

It is well-known that early adverse stress can exert a harmful influence on brain development, behavior and neuroplasticity and lead to psychopathology and cognitive impairments that can persist until adulthood. The occurrence of mental illnesses is closely associated with adverse experiences in childhood (Saleh et al., 2017). In addition, some research has suggested that adults with major depression and ELS are more difficult to treat than those who had not been exposed to ELS (Targum and Nemeroff, 2019). The aim of the present study was to explore whether MS during early life can aggravate the negative impact of stress later in life. This study showed that ELS increased susceptibility to stress later in life and induced more serious anxiety-like symptoms and cognitive impairments. Moreover, our results suggested that MS decreased the expression of PSD95, synaptophysin, phospho-mTOR, and phospho-s6 in the mouse hippocampus.

### Maternal Separation Increased Anxiety-Like Behavior and Impaired Recognition Function

It has been reported that maternally separated rats showed anxiety- and depressive-like behaviors in adulthood (Fodor et al., 2012; Vetulani, 2013). Many previous studies have reported that CRS induces anxiety and depression in rodents (Campos et al., 2013; Jangra et al., 2020). Therefore, the CRS model has been widely applied in the study of mental illness. In this study, increasing anxiety-like behaviors in the CRS group were also observed. However, it has rarely been reported whether MS exaggerates the anxiety-like behavior of animals following CRS. In present study, we used the OFT and EPM test to assess the anxiety-like behaviors of mice and showed that mice in the MS + CRS group displayed more anxiety-like behavior than those in the CRS group, as evidenced by decreased time spent in the open arms of the EPM and central area during the OFT. This study not only found that CRS induced anxiety-like behavior but also suggested that MS exacerbated the negative effect of CRS. These changes suggested that MS exposure increased sensitivity to stress and led to more serious anxiety-like behaviors than non-exposure to MS in mice. These results concur with clinical research data showing that exposure to early adverse experiences increases the probability of developing anxiety and depression later in life (Heim and Nemeroff, 2001). Thus, ELS aggravates anxiety-like behavior and increases vulnerability to stress later in life.

On the other hand, previous studies have reported that after a protocol of ELS, recognition in animals is impaired (Aisa et al., 2007; Ivy et al., 2010). We found that mice subjected to MS and restraint stress displayed cognitive impairments in the NORT and MWM tests, but not in the CRS group. More specifically, the MS + CRS group was significantly different compared to the control group, but no significant difference in spatial acquisition learning and memory in the MWM was observed between the CRS and control mice. The NORT data also showed that the RI value in the MS + CRS group was lower, indicating that non-spatial learning and memory were also disrupted, although this was not the case in the CRS group. Those results suggested that MS can impaired recognition function. But some studies have claimed that MS does not affect spatial memory in the MWM or that spatial memory was enhanced (Enthoven et al., 2008; Banqueri et al., 2017). These differences may be caused by different MS protocols and animal strains. In addition, no significant disruption in learning and memory was observed in the mice of CRS group in our study, which is in disagreement with previous studies using CRS model (Abidin et al., 2004; Perez et al., 2018). In our present study, we applied chronic restraint stress procedure at adolescence rather than at adulthood. Some reports revealed that effects of adolescent-stress may be delayed (Isgor et al., 2004), which is different from adult-stress. Therefore, we speculated that the delayed effect of adolescent-stress may be the reason for no significant deficits in cognitive functions in CRS group mice compared to control, but it does not mean that CRS has no effect on recognition function.

### Maternal Separation Downregulates the Level of Synaptic Proteins

It has been reported that PND2–PND21 is a key period for hippocampal development. Exposure to ELS during this period may lead to long-term changes in synaptic plasticity (Derks et al., 2016). Synaptic plasticity in the hippocampus is closely related to learning and memory and the onset of multiple mental illnesses, including depression and anxiety (Duman et al., 2016). Reduced synaptic plasticity is not conducive to appropriate adaptive responses to subsequent stress. Promoting synaptic plasticity in the hippocampus can improve spatial learning and memory (Li et al., 2019). It is well-known that impairments in synaptic plasticity are associated with the downregulation of synaptic proteins. Therefore, we detected the levels of synaptic proteins in presynaptic and postsynaptic membranes, including PSD95 and synaptophysin, which are closely related to synaptic plasticity. The experimental results showed that the mRNA and protein expression levels of PSD95 and synaptophysin in the hippocampus were lower in the MS + CRS mice than in the CRS mice. These results revealed that MS reduced synaptic proteins and had a negative effect on synaptic function and plasticity. This finding is in line with previous studies showing a downregulation in PSD95 protein expression in maternally separated rats (Ganguly et al., 2015). Thus, this finding suggests that anxiety-like behavior and recognition deficits in maternally separated mice are related to down regulation of the expression of hippocampal synaptic proteins.

### Maternal Separation Inhibits the mTOR-S6 Pathway

In the present study, MS significantly reduced synaptic protein levels of PSD95 and synaptophysin. It has been confirmed that MS leads to a deficiency in synaptic protein translation and has detrimental effects on localized *de novo* activity-induced synaptic protein translation (Ahmad et al., 2018). Alterations in synaptic protein translation are regarded as important facets of neuronal pathologies and neuropsychiatric disorders (Li et al., 2010; Wang et al., 2010). The activity of mTOR regulates translation initiation. Definitive evidence has suggested that mTOR is closely related to the process of synaptogenesis. Activation of mTOR leads to phosphorylation of s6, thereby upregulating the expression of PSD95 and synaptophysin and promoting the production of new synapses (Duman et al., 2012). The mTOR-s6 pathway can potentiate synaptic transmission (Luft et al., 2004) by facilitating the synthesis of synaptic proteins (Bhattacharya et al., 2012). Activation of the mTOR-s6 pathway contributes to synthesis of postsynaptic protein proteins (Tavares et al., 2018). Antidepressants activate the mTOR/s6 kinase signaling pathway and increase the expression of synaptic proteins (Tavares et al., 2018). In this study, the activation of mTOR-s6 was investigated to determine the underlying molecular mechanisms. Our results showed that the levels of phosphorylated mTOR and s6 were significantly decreased in the hippocampus of MS + CRS mice compared to CRS and control mice. This shows that ELS reduced mTOR-s6 signaling activity in the hippocampus.

In summary, we speculate that the mechanisms underlying synaptic plasticity following MS are possibly associated with mTOR-s6 signaling. Notwithstanding, this study has certain limitations. The effects of mTOR inhibitors, such as rapamycin, were not evaluated in the present study. Thus, additional work needs to be done to address these limitations in the future.

## Conclusion

In conclusion, we found that maternal separation aggravated anxiety-like behavior and cognitive deficits and disrupted synaptic plasticity. The mechanisms of these effects may be related to mTOR-s6 pathway inhibition. This implies that clinical treatments of individuals exposed to early-life stress should be different from those without early adverse experiences.

## Data Availability Statement

All datasets generated for this study are included in the article/Supplementary Material.

## Ethics Statement

The animal study was reviewed and approved by the Institutional Animal Care and Use Committee at the Jinan University (Approval No. IACUC-20190702-03).

## Author Contributions

AW, JC, and XZ conceived and designed the experiments. AW and XL performed the research and wrote the manuscript. JW and NY contributed to the animal experiments. AW, QM, and XL contributed to the conducted molecular experiments. NY, JW, and XZ analyzed the data. JC funded the research and revised the manuscript. All authors read and approved the final manuscript.

## Conflict of Interest

The authors declare that the research was conducted in the absence of any commercial or financial relationships that could be construed as a potential conflict of interest.
