# Supplementary figures and images for: Early-Life Stress Alters Synaptic Plasticity and mTOR Signaling: Correlation With Anxiety-Like and Cognition-Related Behavior
Source: Front Genet. 2020 Dec 14;11:590068. doi: 10.3389/fgene.2020.590068 (PMC7767996; doi:10.3389/fgene.2020.590068)

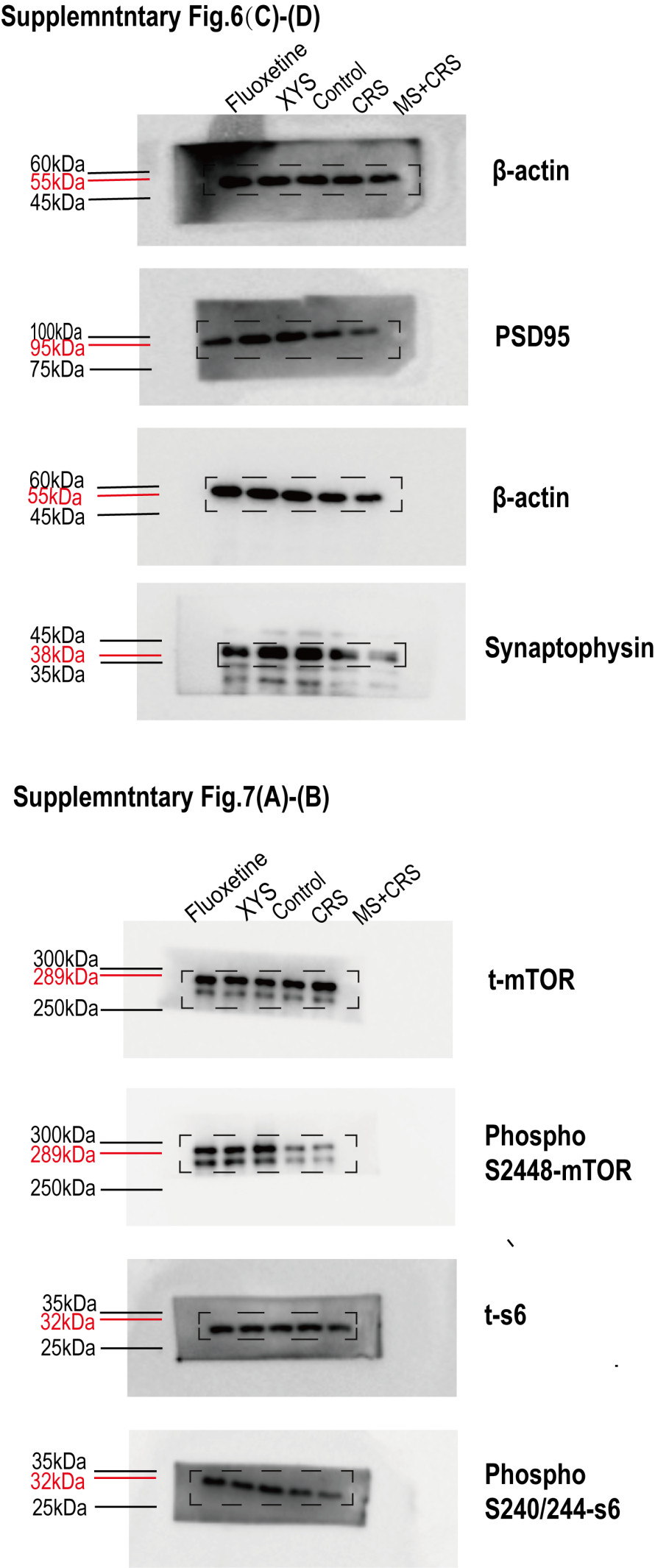

Supplement: Supplementary file 1 [file Data_Sheet_1.DOCX]
