# Supplementary material for: Early-Life Stress Alters Synaptic Plasticity and mTOR Signaling: Correlation With Anxiety-Like and Cognition-Related Behavior
Source: Front Genet. 2020 Dec 14;11:590068. doi: 10.3389/fgene.2020.590068 (PMC7767996; doi:10.3389/fgene.2020.590068)
Supplement: Supplementary file 2 [file Data_Sheet_2.DOCX]

Supplementary Material

**Early-life stress alters synaptic plasticity and mTOR signaling: Correlation with anxiety-like and cognition-related behavior**

Anfeng Wang^1^,Xiaojuan Zou^1^, Xiaojuan Li^3^, Jiajia Wu^2^, Qingyu Ma^3^, Naijun,Yuan^3^,Fengmin Ding^1^, Jiaxu Chen^2,3*^

1 School of Basic Medical Science, Hubei University of Chinese Medicine, Wuhan, Hubei 430065, China.

2 School of Basic Medical Science, Beijing University of Chinese Medicine, Beijing 100029, China.

3 Formula-pattern Research Center, School of Traditional Chinese Medicine, Jinan University, Guangzhou 510632, China.

Corrsepondence：

Jiaxu Chen

[chenjiaxu@hotmail.com](mailto:chenjiaxu@hotmail.com).

# Supplementary Figures

# In my manuscript,images of blots are shown in figures6-7 are not enhanced or obscured.

# The full scan of the entire original gel(s) include five groups：contol,CRS,MS+CRS and two treament groups（Fluoxetine and XYS）.But only contol,CRS,MS+CRS group are reported in this manuscript.A complete scan of the original gels are shown in the figure below. If images still do not comply with the requirements,I will repeat experiment of three groups（control,CRS,MS+CRS group).
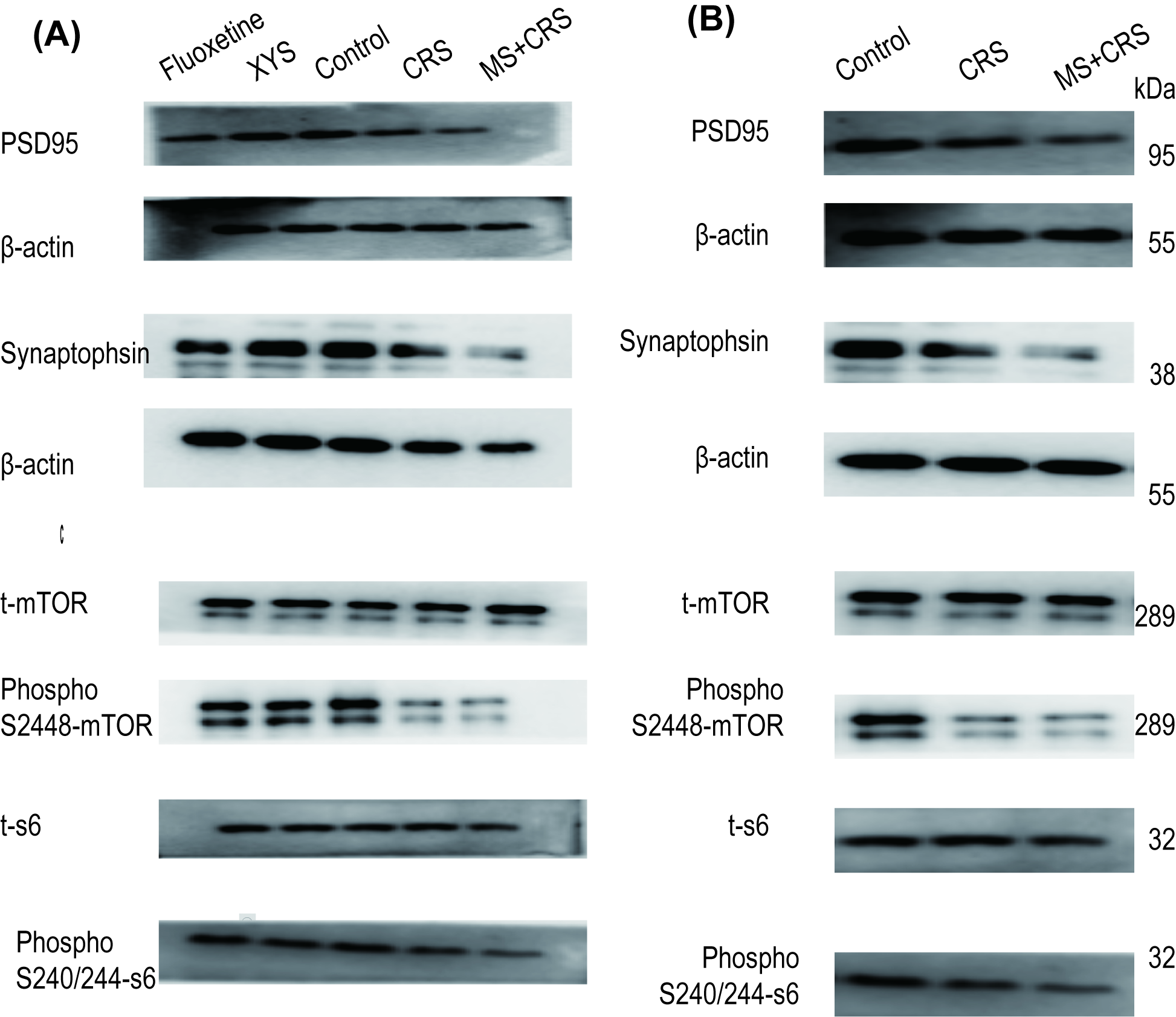
Figure. (A) The full scan of the entire original gel(s) of five groups.(B) Images of blots reported in the manuscript(figures6-7 in the manuscript).
